# Plural Conjuncts and Syncretism Facilitate Gender Agreement in Serbo-Croatian:Experimental Evidence

**DOI:** 10.3389/fpsyg.2019.00942

**Published:** 2019-05-07

**Authors:** Ivana Mitić, Boban Arsenijević

**Affiliations:** ^1^Department of Serbian Language, University of Niš, Niš, Serbia; ^2^Institute for Slavic Languages, University of Graz, Graz, Austria

**Keywords:** agreement, syncretism, gender, number, Serbo-Croatian

## Abstract

The literature on agreement in South Slavic generalizes that conjunct agreement in gender is only possible when all conjuncts are plural (e.g., Bošković, 2009). Marušič et al. (2015) and Arsenijević and Mitić (2016a,b) attest a significant level of patterns contradicting this claim in elicited production experiments. They weaken the earlier generalization to a facilitating role of plural number for conjunct agreement in gender. However, the stimuli in the two respective experiments involve syncretism between the members of conjunction. The syncretism removes the possibility – at Phonological Form at least – that by agreeing with one conjunct, the verb disagrees with the other. It is hence expected to result in a similar surface effect as the facilitation by plurals, which makes it a potential confound variable. We report and discuss the results of an experiment aimed to test both the effect of syncretism and the reality of the facilitating effects of plural number. The results of the experiment yield positive answers to both questions: syncretism is a facilitating factor, but plural number nevertheless has its facilitating effect too – as confirmed by the stimuli without syncretism. Since syncretism is a phenomenon in which phonological information plays a central role, our findings support syntactic models of agreement which extend to the interface with phonology. Moreover, our results reveal a double similarity of conjunct agreement with agreement attraction, in both showing a (stronger) attraction effect of plural number compared to singular, and in being sensitive to syncretism (cf. Badecker and Kuminiak, 2007; Malko and Slioussar, 2013; i.e., Bader and Meng, 2002; Hartsuiker et al., 2003; Slioussar, 2018).

## Introduction

### Relevance of the Research

Grammatical agreement is a hallmark property of human language. Agreement in person, gender, and/or number of features between the subject and the verb is one of its prototypical instantiations. Consider the person and number agreement in the English example in (1).

(1) John smoke-s.vs.John and Bill smoke.

The properties of agreement, especially in conflicting situations, where different (sources of) information can be identified for the same feature, present a highly informative window into the nature of the features and their representation and processing in the brain.

One such conflicting context emerges when the subject consists of two or more conjoined nominal expressions with different number or gender features. What feature does the verb display in such contexts? Does it agree with one of the conjuncts (yielding what is referred to as conjunct agreement), and with which one, or does it display some other (default) feature? What ending should the verb display in (2)?

(2)

**Table:** 

Flaše	i	ogledala		*Serbo-Croatian*
bottle.FPl	and	mirror.NPl		

su	izbačen-?.
AuxPl	thrown.out-?^1^

“The bottles and the mirrors have been thrown out.”

Sometimes, the conjuncts within the subject have different values of number and gender, but these combinations have phonologically identical exponents (a phenomenon known as syncretism) – leading to an even more complex situation. If the ending on the verb in (3) were -*a*, would it stand for FSg, NPl, or would it be underspecified between them?

(3)

**Table:** 

Flaša	i	ogledala	*Serbo-Croatian*
bottle.FSg	and	mirror.NPl		

je/su	izbačen-a.
AuxSg/Pl	thrown.out-?

“The bottles and the mirrors have been thrown out.”

Here, the suffix -*a* stands in one case for the combination FSg, and in the other for NPl. In neither of the two occurences is it possible to identify the individual realizations of number and gender: the two features have a so-called fused realization.

The present research looks into this type of construction: verbs agreeing with a conjunction of two nouns with different number and gender features characterized by a fused syncretic realization, and informs two questions about syntactic features:

I. Does a fused morphological realization of two features by one simplex affix, in this case number and gender, imply that they are also computed as a bundle, or are they rather separately computed features bound by certain dependency relations?
II. Does syncretism in the morphological realization of combinations of different values of a set of features affect their processing in agreement?

Both these questions have theoretical linguistic as well as psycholinguistic relevance. In theoretical linguistics, they have been investigated for a wide range of languages, from Arabic (e.g., Aoun et al., 1994), to Hindi (e.g., Bhatt and Walkow, 2013), and to Slavic (e.g., Bošković, 2009), with a rich body of literature discussing the theoretical consequences of these facts (McCloskey, 1986; Munn, 1999; Doron, 2000; Citko, 2004, among many others).

In psycholinguistics, the question of the bundled vs. independent representation of number and gender has been investigated a.o. in Vigliocco et al. (1996), De Vincenzi (1999), De Vincenzi and Di Domenico (1999), Faussart et al. (1999), Igoa et al. (1999), Hinojosa et al. (2003), Barber and Carreiras (2005), Carminati (2005), Nevins et al. (2007), and Fuchs et al. (2015). Syncretism has been observed to play a role in agreement attraction – a process whereby the target of agreement displays the features of an unexpected expression referred to as the attractor. Typically, this is a nominal expression which intervenes in the linear order between the grammatical controller (by default, the subject) and the target (*the verb*). Consider example (4), where instead of the singular feature of the subject (*the box*), the verb receives the plural feature of the attractor (*the books*).

(4) The box with the books are in the basement.

The more features an expression shares with the controller, the more likely it is to act as an attractor. Syncretism between the controller and the attractor is one such similarity: it has been observed that having an ending syncretic with the ending of the grammatical controller of agreement increases the chances an expression will attract agreement (Bader and Meng, 2002; Hartsuiker et al., 2003; Slioussar, 2018). Moreover, plural number has been shown to be a stronger attractor than singular (Badecker and Kuminiak, 2007; Malko and Slioussar, 2013) – which makes for another parallel with the attractive power of the plural number on conjuncts in competition with singular.

### Mixed Agreement in Gender and Number: Empirical Facts and Theoretical Relevance

Both the traditional and formal literature on conjunct agreement in Serbo-Croatian (henceforth SC), from Maretić (1899) to Bošković (2009), draw the empirical generalization that agreement in gender with a single conjunct obtains only when all conjuncts are plural (Pl).^2^ In other cases – whether with all singular (Sg) conjuncts, or with a combination of Sg and Pl – mixed gender conjunction triggers default agreement (MPl). The empirical picture as reported is illustrated in (5).

(5)

**Table:** 

a.	Flaše	i	ogledala	su	*SC*
	bottle.FPl	and	mirror.NPl	AuxPl	

		izbačen-e/izbačen-a/izbačen-i.
		thrown.out-FPl/-NPl/-MPl^3^

		“The bottles and the mirrors have been
		thrown out.”

b.	Flaša	i	ogledalo	su
	bottle.FSg	and	mirror.NSg	AuxPl

		^∗^izbačen-e/^∗^izbačen-a/izbačen-i.
		thrown.out-FPl/-NPl/-MPl

		“The bottle and the mirror have been
		thrown out.”

c.	Flaša	i	ogledala	su
	bottle.FSg	and	mirror.NPl	AuxPl

		^∗^izbačen-e/^∗^izbačen-a/izbačen-i.
		thrown.out-FPl/-NPl/-MPl

		“The bottle and the mirrors have been
		thrown out.”

Arsenijević and Mitić (2016a,b) present experimental evidence that this is not entirely correct, and that with all Sg conjuncts – gender agreement in SC may still target a single conjunct. They report a significant level of production, as well as an only partial degradation of acceptability of sentences like (5b) when the verb agrees in gender with the first or with the last conjunct (henceforth First Conjunct Agreement, shorter FCA, and Last Conjunct Agreement, shorter LCA), suggesting that (6) is a more accurate empirical report than (5b).

(6)

**Table:** 

Flaša	i	ogledalo	su	*SC*
bottle.FSg	and	mirror.NSg	AuxPl

^(?)^izbačen-e/^(?)^izbačen-a/izbačen-i.
thrown_out-FPl/-NPl/-MPl

“The bottle and the mirror have been thrown out.”

With FCA or LCA in gender, examples of this type manifest mixed agreement: agreement where gender has a single conjunct as a control, while number takes plural – either as the value of the entire conjunction, or as the semantically default value, but crucially a value that is not represented on any of the conjuncts.^3^ This pattern has been observed also on combinations of conjuncts of different number (Sg and Pl) in Slovenian (Marušič et al., 2015: 25–26), a language with very similar behavior to SC when it comes to conjunct agreement. Their study is also the first study in South Slavic conjunct agreement that examines the behavior of doubly mixed conjunctions: those where the conjunct share neither the value for gender, nor for number (in particular, the combinations of neuter singular and feminine plural, and of neuter plural and feminine singular were examined: NSg&FPl, FPl&NSg, NPl&FSg, FSg&NPl).

### Theoretical Modeling of Number, Gender, and Agreement in These Two Features

The investigation reported and discussed in the present paper targets the empirical issues of the effect of syncretism on agreement and of the attracting power of the plural number for agreement in gender. It has consequences for the question whether number and gender are represented as one feature-bundle or separately, and whether they enter agreement together or apart. It also has consequences for the question of whether agreement extends to the syntax–phonology interface. Further than that, it does not directly bear on any particular analysis or theoretical model of the representation of gender and of the operation of agreement. But in the interest of a better understanding of the phenomena discussed, and their theoretical relevance, we briefly present a somewhat simplified model of gender and number representation and agreement.^4^

At least since Ritter (1993), models have been entertained in which gender and number are syntactically represented separately, in two different projections within the nominal domain. Ritter argues that in languages where the grammatically relevant feature is gender itself, as in Hebrew, it figures as a feature of the nominal lexical category head with a derivational value (it derives a noun from another word or from the root), as in (7a), while in those where the relevant nominal property is rather the declension class (or the “word marker,” as she calls it), as in Romance, this property is represented as a feature on number, in NumP, as in (7b).


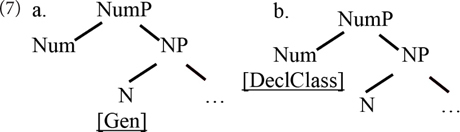


Both views lend themselves well to analyses arguing for an attraction effect of number regarding gender agreement. Assuming that the verb searches (probes) the local structural domain for number and gender features, obeying certain structural restrictions (as per Chomsky, 2001), in the structure in (7a), the search will come across number before reaching gender – as graphically represented in (8). The value of number encountered can influence how agreement proceeds. An effect in the opposite direction is predicted to be impossible to obtain.


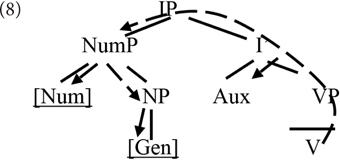


The long-dashed line with arrows represents the direction of search for a gender and number feature.

The structure in (7b) is even more straightforward: declension class is a feature residing on number, and therefore is expected to be sensitive to the narrow value of number. In this case, however, dependencies in the opposite direction are not excluded either.

In the meantime, arguments have been provided that even in Romance, number and declension class are represented separately. Fuchs et al. (2015) provide experimental evidence for a separate representation of number and gender in Spanish.

Serbo-Croatian is a language in which what is referred to as gender agreement is sensitive to both the semantic gender and the declension class of the noun [see Bošković (2009) for an argument that the two behave differently regarding conjunct agreement]. Findings like those in Arsenijević and Mitić (2016a,b), illustrated in (6) above, suggest that in SC the relevant features are specified separately from number – and it is exactly the reliability of these findings that are tested in the present paper.

A hierarchical ordering similar to that in (7a) obtains with coordinated subjects. Conjunction of nominal expressions is known to derive semantically plural referents [but see Heycock and Zamparelli (2005) for a somewhat more complex view]. This can be modeled in terms of a plural feature in the conjunction phrase (ConjP, also referred to in the literature as the Boolean phrase, BoolP).^5^ As illustrated in (9), conjunction itself has no effect on the interpretation of gender. Moreover, at least when the conjuncts are of different gender values, there is no single gender value that can be specified on the ConjP.Again, number – in this case plural – ends up hierarchically more local to the verb, and therefore with the capacity to trigger attraction effects regarding gender.


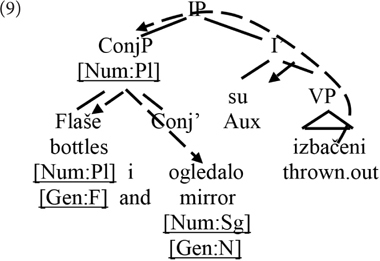


A range of different accounts of agreement have been proposed in the literature. Analyses of conjunct agreement in SC can be roughly classified in two families. One considers agreement a purely syntactic phenomenon. Bošković (2009), or Puškar and Murphy (2015), only use the syntactic operations Merge, Move (including pied-pipe), and Agree to derive the empirically attested patterns and eliminate the ungrammatical ones, exclusively relying on hierarchical structures, in particular on hierarchical locality, illustrated in (10a) for the relevant structural positions (by the underlined specification in the form = N, where the N component specifies the relative locality of the node to the verb from which the search originates). Marušič et al. (2007, 2015), on the other hand, argue that the linear locality of a conjunct to the verb is the strongest factor in Slovenian, a close relative of SC. They propose an account in which in agreement involves a crucial role of the interface with phonology, at which point linear locality plays an important role. Linear locality is illustrated in (10b).


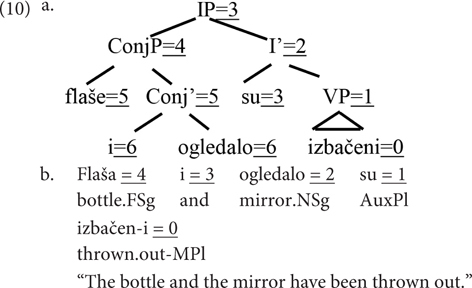


The purely syntactic accounts have the locus of complexity in the syntactic operations involved in agreement (a complex interaction of different syntactic operations determines agreement), but avoid involving phonological considerations. The accounts involving a role of the interface with phonology place the complexity at the modular level, while dealing with simpler structural relations (agreement is determined by plain hierarchical and/or linear locality). Rather than resorting to complex computations within the module of syntax, they distribute them between two modules: syntax and phonology, with relatively simple computations within each, but with two modules involved rather than only one.

### Marušič et al. (2015) Model of Conjunct Agreement

As noted in the section “Relevance of the research,” effects that can be explained as attraction exhibited by the value of number specified on a conjunct over the gender agreement with that conjunct are observed in SC and in Slovenian. In order to account for them, while still deriving mixed agreement (referred to in their article as partial agreement), Marušič et al. (2015: 25–26) state the generalization that “[mixed] Agreement in Gender is allowed only when the Agreement value registered by the targeted conjunct Cx matches the Number value already on the verb (acquired from [ConjP])”^6^ and argue for the following agreement procedure:

**Table:** 

Step 1a.	Agree: Participle Number([ConjP])
Step 1c.	Copy-value: Participle Number([ConjP])
Step 2a.	Agree: Participle Gender([ConjP]) → No Value on [ConjP]
Step 2b.	Choose a Conjunct Cx where Number(Cx) = Number(Participle) Agree: Participle Gender(Conjunct Cx)
Step 2c.	Copy-Value: Participle Gender(Cx)

We illustrate this in (11), on the example originally introduced in (2).

(11)

**Table:** 

[_ConjP_ Flaše	i	ogledala]	*SC*
bottle.FPl	and	mirror.NPl	
su	izbačen-a.
AuxPl	thrown.out-NPl

“The bottles and the mirrors have been thrown out.”

**Table:** 

Step 1a.	*Agree: Participle Number(ConjP)* In this step, the ConjP (the conjoined subject) is simply marked as the source of the number feature to occur on the participle.
Step 1c.	*Copy-Value: Participle Number(ConjP)* In this step, the number feature of the ConjP is copied onto the verb. ConjP is plural, since it involves two conjoined members (*bottles* and *mirrors* – that they are also plural only strengthens the plural status of the ConjP), hence the participle also receives the plural value.
Step 2a.	*Agree: Participle Gender(ConjP) → No Value on ConjP* In this step, ConjP is marked as the source of the gender feature to occur on the participle. However, no such feature is specified on ConjP due to the conflict among the gender values of the conjuncts (the first conjunct is feminine, the second is neuter).
Step 2b.	*Choose a Conjunct Cx where Number(Cx) = Number(Participle)* *Agree: Participle Gender(Conjunct Cx).* In this step, one conjunct is found, which matches the already copied value of number on the participle, and it is marked as the source of the gender to occur on the participle – in the example above, it is the last conjunct.
Step 2c.	*Copy-Value: Participle Gender(Cx)* In this step, the gender feature of the last conjunct is copied onto the verb. This conjunct is neuter, hence the participle also receives the neuter value.

The verb first agrees in number with the entire conjunction, thus receiving the value plural. Then it attempts to agree in gender with the entire conjunction – but fails since the ConjP is unspecified for a gender value due to the mixed gender values of its conjuncts. It then attempts to agree with the most local conjunct (in some grammars hierarchical locality matters, yielding FCA; in others linear locality, yielding LCA). However, conjunct agreement is not free – it is conditioned by the identity of the number value already acquired by the verb and the number value on the targeted conjunct. Since the value already acquired by the verb is plural, then as a result, plural number on the conjunct facilitates gender agreement with that conjunct.

A similar view is advocated by Arsenijević and Mitić (2016b), who investigate agreement with conjoined singulars. They observe that even singular agreement is attested on the verb at significant rates. As this pattern is unexpected on Marušič et al. (2015) model, where the verb must acquire the plural value of number, Arsenijević and Mitić (2016b) offer an alternative based on three soft constraints:

1. The verb should agree in number with the entire conjunction,
2. The verb should agree in gender with the local conjunct, and
3. The verb should agree with the same constituent in both gender and number.^7^

Plural conjuncts are more likely gender-agreement controllers than singular conjuncts because they allow for plural number on the verb to be interpreted both as agreement with the entire conjunction (hence avoiding a violation of the constraint 1 above) and as agreement with the plural conjunct (thus avoiding a violation of the constraint 3 above). With singular controllers of gender, if the verb is singular, it does not agree with the entire conjunction (violating constraint 1 above), and if it is plural, it does not have the same control as gender (violating constraint 3 above). In both cases, one of the constraints gets violated, and it is the ordering of constraints that decides the winner. On this view, plural number on the conjunct facilitates agreement in gender because when the verb agrees in gender with a plural conjunct – it satisfies both the constraint that it matches the number of the controller of gender agreement, and the one that requires it to match the number on the ConjP.

Both these investigations suffer from failing to control for one potential confound variable which is expected to have effects similar to those reported. Since masculine is the default gender in South Slavic conjunct agreement, in order to clearly attest FCA and LCA, the conjuncts must bear a combination of a feminine and a neuter gender value. In that case, each of the three gender values can in principle occur on the verb and signal a different agreement pattern: feminine and neuter the two different patterns of conjunct agreement, and masculine the default agreement. Both investigated languages, Slovenian and SC, display syncretism between FSg and NPl [compare (12a vs. 12d)], as well as between FPl and about a half of NSg nouns [compare (12b vs. 12c)]. This substantially undermines the findings of these two investigations: it is possible that the mixed agreement is simply an effect of the syncretism.

(12)

**Table:** 

a.	žen-a	knjiga	slik-a	*SC*
	woman-FSg	book-FSg	picture-FSg
	stolic-a
	chair-FSg
b.	žen-e	knjig-e	slik-e	stolic-e
	woman-FPl	book-FPl	picture-FPl	chair-FPl
c.	sel-o	let-o	polj-e	mor-e
	village-NSg	summer-NSg	field-NSg	sea-NSg
d.	sel-a	let-a	polj-a	mor-a
	village-NPl	summer-NPl	field-NPl	sea-NPl

In Marušič et al. (2015), syncretism may be facilitating the ending that phonologically matches both conjuncts [the ending -*e* on the verb in (13a)]. In Arsenijević and Mitić (2016b) it is possible that the ending on the verb is supported by its phonological match with one conjunct in the form used, and with the plural form of the other [see (13b), where the feminine conjunct has *zakletv-e* as its plural form, and the neuter conjunct *obećanj-a*].^8^ Since the verb tends to, or must be plural – it is reasonable to expect that this latent syncretism also plays a role. Especially considering that if the verb were plural and agreed in gender with one singular conjunct, its ending would be syncretic with that on the other singular conjunct. Therefore, in the results of both Marušič et al. (2015) and Arsenijević and Mitić (2016a,b), when the verb receives the ending -*e* or the ending -*a*, it is impossible to reliably determine whether it only does it due to bearing the respective features (and which features: NPl or FSg?), or it is, partially at least, because it phonologically matches the ending on one or both of the conjoined nouns.^9^

(13)

**Table:** 

a.	Tel-e	in	krav-e	so	*Slovenian*
	calf-NSg	and	cow-FPl	AuxPl

	se	skril-e/skril-a	za	grmièevje.
	Refl	hid-FPl/NPl	behind	shrubs

	“The calf and cows hid behind the shrubs.”

b.	Zakletv-a	i	obećanj-e	su	*SC*
	oath-FSg	and	promise-NSg	AuxPl

	prekršen-e/prekršen-a.
	broken-FPl/NPl

	“The oath and the promise have been broken.”

### Hypotheses and Predictions

The null hypothesis predicts that the three types of agreement, FCA, LCA, and DEF, will be equally represented in the results, both with and without syncretism. However, since substantial research has already been done on some of the variables that have been controlled in our experiment, we can formulate a more informed, and more relevant, relative null hypothesis – as well as several competing alternative hypotheses and their predictions.

The reports in the literature before Marušič et al. (2015) and Arsenijević and Mitić (2016a,b) predict that due to the different number values on the conjuncts, only DEF will be produced. A significant level of production of FCA and/or LCA in gender would reject this view.

**FIGURE 1:**
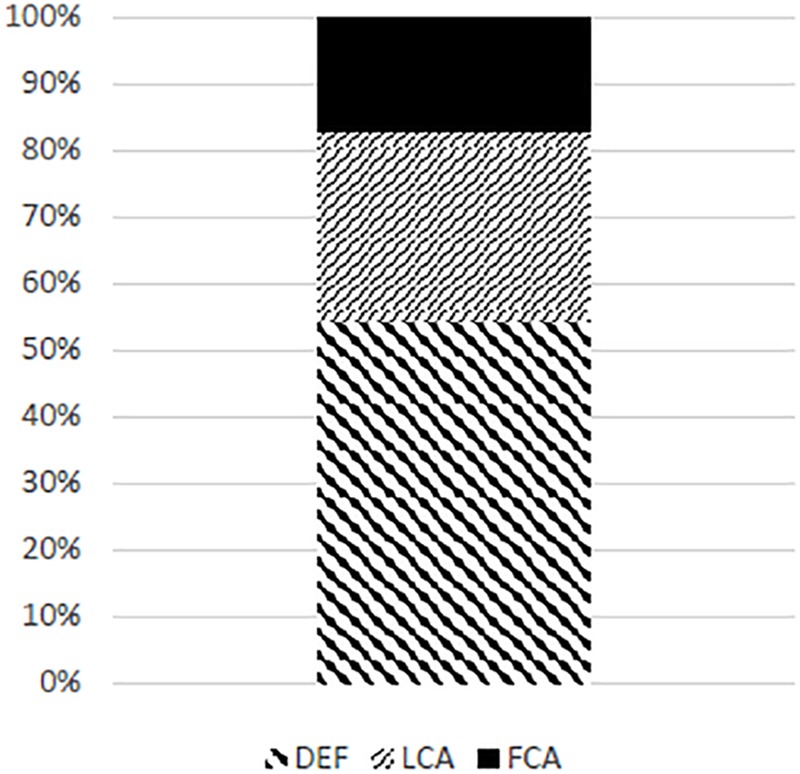
The ratio between DEF, LCA, and FCA for all plural conjuncts.

Hypotheses predicting conjunct agreement in gender need to be informed about the general ratio between the three agreement strategies, FCA, LCA, and DEF, in the configurations in which they are not suppressed or asymmetrically facilitated by additional factors. The best candidate for such a configuration is one with coordinated subjects involving only plural conjuncts. Willer-Gold et al. (2016, 2018) show that with this type of conjoined subjects, when the first conjunct is neuter and the last is feminine – DEF is the strongest strategy, followed by LCA – with FCA as the least produced pattern.^10^ This is shown in Figure 1.

These results can be taken as base-line expectations for the gender combination F&N, if we accept the conclusion based on the reports in Marušič et al. (2015) and Arsenijević and Mitić (2016a,b), namely that the agreement in mixed number and gender conjunctions is a special case of mixed gender conjunction agreement, with an additional facilitating effect of the plural number. Deviations from the distribution in Figure 1 in that case indicate the effects of the two factors that we are investigating: facilitation of conjunct agreement in gender by the plural value of number and syncretism. This allows us to formulate the following alternative hypotheses and their predictions.

**Hypothesis 1**: As suggested in the literature (Bošković, 2009), a mixed value of number triggers DEF in number, there is no effect of syncretism whatsoever.
**Prediction 1**: In both conditions, only DEF will be produced, with zero instances of either FCA or LCA.
**Hypothesis 2**: Plural facilitates gender-agreement with the conjunct that bears it, because it matches the value of number of the entire conjunction. This hypothesis is an alternative to Hypothesis 1, as it makes the assumption that mixed gender and number conjuncts are a special case of mixed gender conjuncts. Therefore, it takes the production of agreement with all plural conjuncts, given in Figure 1, as a baseline.
**Prediction 2**: The ratio between LCA and FCA will change in favor of LCA in the condition without syncretism, in comparison to the base-line ratio in Figure 1.
**Hypothesis 3**: Syncretism facilitates conjunct agreement because the verb can then be interpreted both as showing FCA and LCA.
**Prediction 3a**: Combined with Hypothesis 1, it predicts that the condition without syncretism will elicit only DEF, with zero FCA and LCA, while the condition with syncretism will possibly elicit some LCA in addition to DEF.
**Prediction 3b**: Combined with Hypothesis 2, it predicts that on top of the effect of plural-facilitation (more LCA, less FCA in both conditions), syncretism will cause an additional increase of LCA and at the expense of DEF compared to the non-syncretic condition.

We conducted an experimental study to test whether indeed syncretism facilitates the production of the respective endings on the verb. Controlling for syncretism allowed us to examine our central question, i.e., to test whether the facilitation effect of the plural number on one of the conjuncts is real regarding the production of mixed agreement.

In the section “Elicited Production Study,” we report and discuss the design and results of this experiment. The section “Design and Materials” describes the methodology, the experimental material used, and the fitting of the design, and the section “Participants” provides the information about the participants. The section “Procedure” summarizes the competing generalizations and hypotheses, and their predictions, and section “Procedure” reports the results. In the section “Results,” we discuss how the results bear on the predictions outlined in the section “Procedure.” The section “Conclusion” is the conclusion.

## Elicited Production Study

### Design and Materials

In order to investigate the effect of syncretism and facilitation of conjunct agreement by plural number in SC, we have designed and conducted an elicited production experiment, adopting the methodology implemented and reported in Marušič et al. (2015), Arsenijević and Mitić (2016a,b), Willer-Gold et al. (2016, 2018), and Mitić and Arsenijević (2019) and several other experimental works. The experiment was developed and administered using the Internet portal Ibex Farm^11^.

#### Independent and Dependent Variables Adopted

We only had one dependent variable: the gender agreement pattern produced, with three levels: FCA (N), LCA (F), and DEF (M). Due to the mixed combination of genders, true resolved agreement (RES) from Willer-Gold et al. (2016), where the aggregate conjunction has the gender value shared by all the conjuncts, was not an option. There was only one manipulated independent variable: the presence vs. absence of syncretism between the conjuncts, i.e., whether the two conjuncts had homophone endings.

#### Properties of the Stimuli

All the sentences had preverbal subjects, were of approximately the same length in syllables and characters (mean length in syllables = 8.83, standard deviation = 0.70, mean length in characters = 26.00, standard deviation = 0.59), and involved nouns of similar frequency (average frequency 0.05 tokens per 1000 words, standard deviation 0.02, as per the Corpus of Contemporary Serbian Language, Krstev and Vitas, 2005)^12^. All the stimuli involved substitute subjects consisting of two conjoined disyllabic bare nouns (SC has no articles, hence bare nouns are fully unmarked), where the first member of conjunction was a NSg noun and the second a FPl noun. All substitute subjects had the identical length in syllables (five syllables each), and their length in characters ranged from 11 to 13, with a mean at 12.75, standard deviation: 0.61.

Out of the four possible combinations (NSg&FPl, NPl&FSg, FSg&NPl, FPl&NSg) – we included only one (NSg&FPl), for two reasons. One was that we wanted to keep as many variables controlled rather than tested, and avoid overcomplicating the experiment. Testing both variables – the order of gender values [shown to be a factor in Arsenijević and Mitić (2016a) and Willer-Gold et al. (2016, 2018)] and the gender value (in particular feminine or neuter) which is combined with the plural value of number [cf. the results in Marušič et al. (2015) for Slovenian] – is a task for further research. The other reason requires more details of the experiment to be introduced, and is elaborated below, and illustrated in (21). All the predicates in the stimuli were passive forms of transitive verbs.

#### The Stimuli

The experiment involved 60 stimuli: 12 critical (6 for each condition) and 48 fillers. The stimuli for each of the two conditions are illustrated in (14).

(14) Illustration examples for the two conditions
  a. Condition with syncretism (both the NSg and the FPl noun end in *-e*):
  Model sentence (i.e., first screen):

**Table:** 

Ručak	je	pojeden	na	brzinu.
lunch.MSg	is	eaten.MSg	on	speed

“The lunch was eaten in rush.”
Substitute subject (i.e., second screen):

**Table:** 

jaje	i	šljive
egg.NSg	and	plum.FPl

b. Condition without syncretism (the NSg noun ends in *-o*, the FPl noun in -*e*)
Model sentence (i.e., first screen):

**Table:** 

Dokaz	je	ukraden	iz	torbe.
evidence.MSg	is	stolen.MSg	from	bag

“The evidence was stolen from the bag.”
Substitute subject (i.e., second screen):

**Table:** 

pismo	i	mape
letter.NSg	and	map.FPl

There were two types of fillers. They were all identical in design like the critical examples (a model sentence with a MSg subject followed by a substitute subject), except that they had different substitute subjects. One group (*N* = 18), illustrated in (15a), involved conjoined substitute subjects with both plural conjuncts: FPl&NPl, such that one or both of the conjuncts were modified by an agreeing adjective. In the other (*N* = 30), the substitutes were nouns with a special behavior regarding number and gender, falling in five different sub-types, each represented with six items, illustrated in (15b–f).^13^ The complete list of the stimuli is provided in the Supplementary Table S1.

(15)

a. *Model sentence (i.e., first screen):*

**Table:** 

konac	je	donet	kod	krojačice.
thread.MSg	AuxSg	brought	at	tailor

“The thread was brought to the tailor’s.”
Substitute subject (i.e., second screen):

**Table:** 

ljubičaste	igle	i	zrna.
violet.FPl	needle.FPl	and	bead.NPl

“violet needles and violet beads”
b. *Model sentence:*

**Table:** 

čuvar	je	obišao	zgradu.
guard.MSg	AuxSg	visited	building

“The guard visited the building.”
Substitute subject:

**Table:** 

Julijin	komšija
Julija’s.MSg	neighbor.FSg/MSg

c. *Model sentence:*

**Table:** 

vlasnik	je	došao	u	pekaru.
owner.MSg	AuxSg	come	in	bakery

“The owner came to the bakery.”
Substitute subject:

**Table:** 

moje	cerekalo
my.NSg	laugher.NSg/MSg

d. *Model sentence:*

**Table:** 

konj	je	trčao	po	polju.
horse.MSg	AuxSg	run	on	field

“The horse ran around the field.”
Substitute subject:

**Table:** 

prvo	prase
first.NSg	pig.NSg

e. *Model sentence:*

**Table:** 

rezultat	je	ohrabrio	studente.
result.MSg	AuxSg	encouraged	students

“The result has encouraged the students.”
Substitute subject:

**Table:** 

uspeh	dekana
success.MSg	dean.GenMSg

f. *Model sentence:*

**Table:** 

kolega	je	zvao	u	podne.
colleague.MSg	AuxSg	called	at	noon

“My colleague called at noon.”
Substitute subject:

**Table:** 

prijatelj	iz	škole
friend.NSg	from school	GenFSg.

### Participants

The experiment was conducted at the University of Niš. Thirty-six native speakers of B/C/S who had spent at least the past 5 years within the area in which this language is spoken participated in the experiment, with 18 per list (age range 19–23, average age 20.61, standard deviation 1.13). Participants included 28 (77.78%) females and 8 males (22.22%). The participants were all students in their first or second year of undergraduate programs which do not involve linguistic courses. A written informed consent was obtained from each participant. An ethics approval was not required for this research as per applicable institutional and national guidelines and regulations.

### Procedure

The experimental procedure involved two steps for each stimulus. In the first step, the participant reads aloud a model sentence involving a masculine singular non-coordinated subject as in (16a), which is displayed on the first screen. In the second step, the second screen shows a substitute subject as in (16b), and the participant pronounces the sentence again, but with the substitute subject instead of the original one – adapting also the morphosyntax of the verb to it.

(16)

a. *FIRST SCREEN*

**Table:** 

ulaz	je	očišćen	prošlog	petka
entrance.MSg	is	cleaned.MSg	last	Friday

“The entrance was cleaned last Friday.”
b. *SECOND SCREEN*

**Table:** 

kupatilo	i	kuhinje
bathroom.NSg	and	kitchen.FPl

The agreement pattern used by the participant in the pronounced sentence is coded as Sg or Pl for number and as FCA, LCA, or DEF (Default) for gender.

The experiment begins with six training examples, used by the administrator to instruct the participants about the experimental procedure. The training examples involved, both in model sentences and as substitutes, only non-conjoined subjects of various, yet balanced number–gender combinations.

The details of the experiment most closely matched the methodology in Mitić and Arsenijević (2019). Critical items were organized in two lists, so that each stimulus occurred exactly once in each condition. The purpose was to control for a possible effect of the particular lexical items, or of various other idiosyncratic properties of the particular stimuli. The lexical items were selected from reference dictionaries, such that the resulting sentences could saliently be used in a natural conversation.^14^ All participants completed the experiment, and were included in the results.

## Results

*Data analysis* was determined by the design of the experiment. Since both the predictor and the dependent variable are categorical, we had originally implemented a χ^2^ test to assess the significance of the relevant differences. One reviewer suggested that we could obtain more reliable insights if we used a linear mixed effects model. Indeed, this test turned out to be partly applicable after we observed that in spite of the principled multi-level nature of the categorical variable of the gender-agreement pattern – the results instantiated only two of the three levels: LCA and DEF, without a single instance of FCA. Effectively, thus, both our categorical variables had two levels, and could be coded as pseudo-scalar variables, where one level is coded as 0 and the other as 1. For the comparisons involving datasets with three levels of the dependent variable (FCA, LCA, and DEF) – we were forced to stick to the χ^2^-test. As the probabilities for all the effects that were significant are of a very low level (*p* < 0.0001 in all of them), we consider the χ^2^-test sufficiently reliable as well.

**FIGURE 2:**
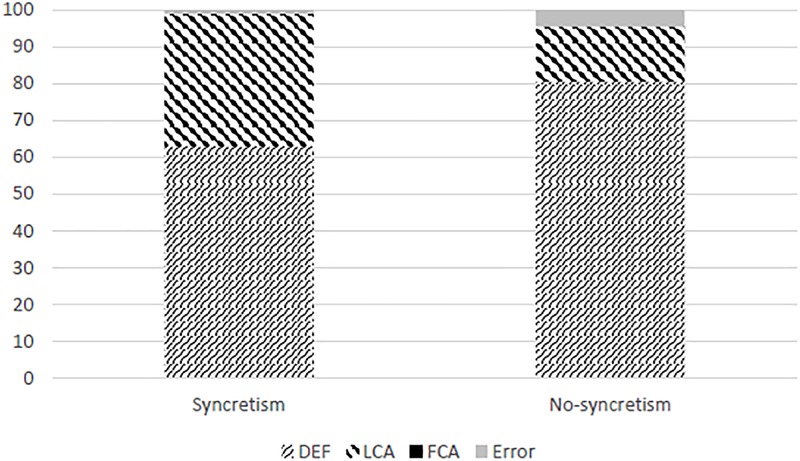
A graphical representation of the produced patterns and errors.

The Results of the experiment, as mentioned above, included only LCA and DEF agreement (see the Supplementary Table S1 for the aggregate raw results). All the produced sentences displayed unambiguous plural number, and no FCA was produced in either condition [i.e., no verbs were produced with the ending -*a*, as in (17d) and (18d), which is ambiguous between FSg and NPl, or with the NSg ending -*o*, as in (17c) and (18c)]. The actual results in percentages are given in Table 1 and graphically represented in Figure 2, followed by illustration examples for each type of result data obtained.

**Table 1:** Results of the experiment.

	DEF (%)	LCA (%)	Error (%)
Syncretism	63	36	1
No syncretism	80.5	15	4.5

(17) Illustration of examples for results in the condition with syncretism
  a. Condition with syncretism, default agreement (63%):

**Table:** 

Jaje	i	šljive	su	pojedeni	na	brzinu. egg.NSg	and	plum.FPl	are	eaten.MPl	on	speed

“The egg and the plums were eaten in rush.”
b. Condition with syncretism, LCA, and/or syncretism (36%):

**Table:** 

Jaje	i	šljive	su	pojedene	na	brzinu.
egg.NSg	and	plum.FPl	are	eaten.FPl	on	speed

“The egg and the plums were eaten in rush.”
c. Condition with syncretism, FCA in number, and gender (0%):

**Table:** 

Jaje	i	šljive	su/je	pojedeno	na	brzinu.
egg.NSg	and	plum.FPl	are/is	eaten.NSg	on	speed

“The egg and the plums were eaten in rush.”
d. Condition with syncretism, Pl, and FCA gender or Sg and LCA in gender (0%):

**Table:** 

Jaje	i	šljive	su/je	pojedena	na
egg.NSg	and	plum.FPl	are/is	eaten.NPl/FSg	on
brzinu.
speed

“The egg and the plums were eaten in rush.”
(18) Illustration of examples for results in the condition without syncretism
  a. Condition without syncretism, default agreement (80.5%):

**Table:** 

Pismo	i	mape	su	ukradeni	iz	torbe. letter.NSg	and	map.FPl	are	stolen.MPl	from	bag

“The letter and the maps were stolen from the bag.”
b. Condition without syncretism, LCA without syncretism (15%):

**Table:** 

Pismo	i	mape	su	ukradene	iz	torbe.
letter.NSg	and	map.FPl	are	stolen.FPl	from	bag

“The letter and the maps were stolen from the bag.”
c. Condition without syncretism, FCA in number, and gender (0%):

**Table:** 

Pismo	i	mape	su/je	ukradeno	iz	
letter.NSg	and	map.FPl	are/is	stolen.NSg	from	
torbe.
bag

“The letter and the maps were stolen from the bag.”
d. Condition without syncretism, Pl, and FCA gender or Sg and LCA in gender (0%):

**Table:** 

Pismo	i	mape	su/je	ukradena
letter.NSg	and	map.FPl	are/is	stolen. NPl/FSg
iz	torbe.
from	bag

“The letter and the maps were stolen from the bag.”

The χ^2^-test has confirmed a significant difference between the distribution of agreement patterns in the two conditions: there was significantly more LCA and less DEF in the syncretic than in the non-syncretic condition [χ^2^(2, *N* = 421) = 22.79, *p* < 0.00001]. Our results hence match the Prediction 2 from the section “Participants”: the effect of syncretism in facilitating non-default agreement is clearly confirmed.

Even though both our variables were categorical (with levels syncretic and non-syncretic for the predictor, and FCA, LCA, and DEF for the dependent variable), due to the absence of FCA observations in the dependent variable both were effectively two-level variables in the data-set. As pointed out by an reviewer, this allows to code them as (pseudo-)scalar variables. We took advantage of this opportunity, and report this test as well. We used the lme4 package (Bates et al., 2012) in R (R Core Team, 2012) to subject the difference between the syncretic and non-syncretic conditions to a linear mixed effects model test. As the predictor we entered presence vs. absence of syncretism (as 0 and 1, respectively), and as the observations for the dependent variables we coded DEF as 0 and LCA and 1. As random effects, we entered items and participants, and we specified the binomial family, without random slopes: *glmer*(*AgreePattern ∼ Syncretism* + (1| *Participant*) + (1|*Item), family = binomial, data = SyncrAgree*). The test confirmed a significant difference between the distribution of agreement patterns in the two conditions (β = -0.205, *t* = -4.897, *p* < 0.0001, where the reference level of the intercept was LCA and the syncretic condition). The absence of syncretism thus resulted in a significantly lower rate of LCA, i.e., there was significantly more LCA and less DEF in the syncretic than in the non-syncretic condition. Our results hence match Prediction 3 from the section “Procedure”: the effect of syncretism in facilitating non-default agreement is clearly confirmed.

In order to assess the significance of *the differences between the two patterns of agreement* produced within conditions, we compared each of the conditions to the null hypothesis regarding the rate of DEF and LCA (i.e., an equal number of elicited sentences for the two patterns). To achieve this, we used the same methodology as above. We pseudo-randomly distributed an equal number of LCA and DEF observations (coded as 1 and 0) across the aggregate number of observations for each level of the predictor variable. Hence as the predictor, we entered the null hypothesis and the relevant condition (i.e., syncretic and non-syncretic in independent applications of the test). We coded them as 0 for the null hypothesis and 1 for the respective condition – syncretic in one application of the test, and non-syncretic in the other). The dependent variable with two levels, DEF and LCA, was again coded as 0 for DEF and 1 for LCA. The linear mixed effects model attested a significant difference between the prediction of the null hypothesis and the result of the experiment for the non-syncretic condition (β = -0.5, SE = 0.03, *t* = -17.64, *p* < 0.0001, Intercept = 0.5). It did not, however, confirm the significance of the difference between the prediction of the null hypothesis and the syncretic condition (β = -0.053, SE = 0.04, *t* = -1.307, *p* = 0.193, Intercept = 0.5). Since syncretism is the marked level, the straightforward interpretation is that the difference between the two patterns of agreement (LCA vs. DEF) is confirmed for conjoined subjects involving conjunct with mixed both number and gender values, but syncretism strengthens LCA to the extent that this difference ceases to be visible.^15^

Even in the non-syncretic condition, there were 15% of produced sentences exhibiting unambiguous LCA. This is a relatively high rate of production, compared with the complete absence of FCA, and with Prediction 1 that no LCA will be produced. Note also that LCA is produced at rates much higher than typical error rates: the level of erroneous productions for this type of task is typically below 5% (as is the case with the clear errors in the present experiment, as well as with the error rates attested in other experiments using similar methodology: Marušič et al., 2015; Arsenijević and Mitić, 2016a,b; Willer-Gold et al., 2016, 2018; Mitić and Arsenijević, 2019).

## Discussion

Our experiment clearly shows that not only is DEF available for conjoined subjects when conjuncts have different number values, but that LCA was present in both conditions (see Table 1 and Figure 2). This clearly rejects the generalizations in the earlier literature, formulated in the section “Procedure” as Hypothesis 1, as well as the hybrid Hypothesis 3a based on the same generalization. A mismatch in number indeed decreases conjunct agreement in favor of default, but it does not eliminate it. Considering the reports of Marušič et al. (2015), Arsenijević and Mitić (2016a,b), and Willer-Gold et al. (2016, 2018) – this decreasing effect probably does not need to be restricted to a mismatch in number, but can also come from a difference in the gender values of the conjuncts within conjoined subjects – which is a topic for a separate investigation.

The results confirm Hypothesis 2, that plural number on a conjunct facilitates agreement with that conjunct, in congruence with the models by Marušič et al. (2015) and Arsenijević and Mitić (2016b). Recall Prediction 2, derived from this hypothesis in the section “Procedure,” that conjoined subjects of the type NSg&FPl used in our experiment will elicit relatively more LCA and less FCA than the all-plural conjuncts in Willer-Gold et al. (2016, 2018); see Figure 1. Our results displayed a significant difference between the condition without syncretism (15% of LCA and 0% of FCA) and the NPl&FPl condition in Willer Gold et al. (30% of LCA and 17.78% of FCA), as well as between the condition with syncretism (36% of LCA and 0% of FCA) and the NPl&FPl condition in Willer Gold et al. Hypothesis 3 from the section “Procedure,” more precisely its version 3b, was also confirmed. The prediction was that the syncretic condition will elicit more LCA and less DEF than the non-syncretic condition, and this difference was attested as significant.

In spite of the negative effect of the double mismatch between the conjuncts, both in gender and in number, the rate of LCA for NSg&FPl was the same or higher than for NPl&FPl subjects in the base-line data-set from Willer-Gold et al. (2016, 2018). The rate of FCA – the condition which was facilitated neither by plural number nor by syncretism, dropped to zero in our experiment, both with and without syncretism. We can conclude that both syncretism and plurals display clear facilitating effects on conjunct agreement in SC.

This means that while syncretism may have been a confounding variable in Marušič et al. (2015) and Arsenijević and Mitić (2016a,b), it was not solely responsible for the results. The generalization that conjunct agreement is not impossible with mixed number conjuncts and that plural on conjuncts facilitates agreement with them was still correct.

A curious question emerges from these results: Why was no FCA at all produced in the present experiment? In the experiment conducted by Marušič et al. (2015), syncretism was not controlled for, but otherwise there is a condition fully matching the type of stimuli in the present experiment: their condition NSg&FPl. This condition yields 5% of produced sentences with FCA. The obvious explanation is that Slovenian and SC are not that similar when it comes to conjunct agreement. Moreover, since the Sg&Sg conjunction tested in Arsenijević and Mitić (2016a,b) also rendered a considerable level of FCA (at the rate of 19%, which is not far from the level of 17% of FCA with Pl&Pl conjunctions reported in Willer-Gold et al. (2016), there seems to be a particularly strong negative effect of the double mismatch in feature values (both number and gender) in SC. Still, no definite conclusion regarding the question why FCA is so strongly suppressed can be offered based on our experiment, and therefore we leave it for further research.

Our results provide support for the models of agreement in which agreement is not a purely syntactic phenomenon, but partly takes place at the interface with phonology [Arregi and Nevins (2012); Marušič et al. (2015), and Willer-Gold et al. (2016, 2018) for South Slavic]. Syncretism is a phenomenon which involves phonological identity of the exponents of different feature–value combinations. If agreement were fully determined by syntactic structure, then syncretism would be less likely to have effect on agreement than if agreement extends to the interface with phonology. In views which distinguish between competence and performance, it is, however, possible that this effect is a matter of performance, and hence orthogonal to the question of modularity of agreement.

This possibility opens up a more general question which has not yet been convincingly answered in the literature: is conjunct agreement a grammatical agreement strategy, or an error similar to agreement attraction? The fact that in conjunct agreement the controller belongs to the subject and carries the relevant morphosyntactic features has made most researchers maintain the former option. This was further supported by the fact that conjunct agreement is produced, and its acceptability is judged, at the levels similar to, or often even significantly higher than those of the agreement plausibly interpreted as targeting the entire ConjP.

The sensitivity of conjunct agreement to syncretism as a property it shares with agreement attraction (Bader and Meng, 2002; Hartsuiker et al., 2003; Slioussar, 2018) calls for reconsidering this view. This is highly compatible with restricting the role of the syntactic structure to narrowing down the retrieval space for the agreement features – while the actual retrieval takes place at the interface with phonology (Arregi and Nevins, 2012; Marušič et al., 2015). Therefore, it is actually expected that those attractors which sit within the narrowest retrieval space will have a sigificantly stronger attraction power – which would explain the higher acceptability and rates of production compared to agreement with attractors which are outside the subject constituent, or with those in peripheral (i.e., modifier) positions within the subject constituent. In this view, an important question is where the line should be drawn between competence and performance within the phonological component of agreement.

Finally, our experiment does not provide decisive evidence for or against the view that number and gender are represented and processed as a bundle, rather than apart. Since in our experiment no FCA was produced (recall that the first conjunct was singular, and the last conjunct was plural) – instances of LCA could be interpreted as LCA in both gender and number, and DEF as default in both features. Counterexamples would be those where number is plural, and gender has the value of the conjunct which has the singular value for number – which were not produced in our experiment (see the Appendix for the reasons we chose the distribution of features NSg&FPl in our experiment). This pattern is more likely to occur when the last conjunct is singular and the first conjunct is plural^16^.

Our experiment provides evidence for a dependence of gender on number, and no such dependency in the other direction. However, as it was not designed to capture the latter, the strongest conclusion we can make in this respect is that number can be processed without gender (no indications regarding the processing of gender without number), and that the processing of gender is dependent on number.

## Conclusion

Two recently proposed models of the interaction of number and gender agreement build on results attesting facilitation of conjunct agreement in gender by a plural value of number on the conjunct. As both experiments that these investigations are based on involve a possible confound variable of syncretism between the conjuncts – we tested both the effect of syncretism, and the facilitation effect of the plural number in the absence of syncretism. Our results are doubly confirming. Syncretism is indeed a factor that facilitates conjunct agreement, but the facilitating effect of the plural is also real. The research lends support to the models of agreement extending to the interface between syntax and phonology, and opens some new questions about conjunct agreement within and between the South Slavic varieties.

## Ethics Statement

As specified in the manuscript, at the time of administration of the experiment, no approval of an ethical committee was required by the positive regulations of the University of Niš or of the Republic of Serbia.

## Author Contributions

Both authors designed the experiment and analyzed the results. IM coded the experiment in Ibex, administered it, and coded the results. BA wrote the manuscript.

## Conflict of Interest Statement

The authors declare that the research was conducted in the absence of any commercial or financial relationships that could be construed as a potential conflict of interest.

## Footnotes

1 The following abbreviations are used in the paper: Aux for *auxiliary*, ConjP for the *conjunction phrase*, F for *feminine gender*, FCA for *first conjunct agreement*, GenP for *gender phrase*, LCA for *last conjunct agreement*, DEF for *default agreement*, M for *masculine gender*, MIX for *mixed agreement*, N for *neuter gender*, N^0^ and n^0^ for the *nominal categorial head*, NP for *noun phrase*, NumP for *grammatical number phrase*, Pl for *plural*, Refl for *reflexive*, SC for *Serbo-Croatian*, and Sg for *singular*.

2 Babić (1998) and Bojović (2003) provide a number of exceptions, but most of their examples involve special kinds of conjunction – that clearly involving ellipsis, that where all the conjuncts after the first conjunct are its appositives, or that where conjunction has a disjunctive interpretation. It is worth noting that the different investigations used different methodologies, and relied on different formats and types of data. Babić (1998) and Bojović (2003) mostly have corpus examples with conjoined subjects of different forms, Bošković (2009) has own examples of the form “one NP and all NPs,” while Marušič et al. (2007, 2015), Willer-Gold et al. (2016, 2018) as well as the present paper observe conjoined bare nouns from the experimental perspective.

3 We refrain from committing to either of the possible analyses of the plural number in these examples: as the value specified on the entire conjunction, labeled ConjP, or as the default value assigned in the absence of a specified value; we refer to it descriptively throughout the paper as default number agreement.

4 Including such an overview was suggested to us by an reviewer, for which we express our gratitude.

5 In approaches like Citko (2004), the plural feature is not in ConjP, but on a pronominal element generated on top of it.

6 They refer to (the projection standing for) the entire conjunction as the BoolP, but it has been replaced here with the notation ConjP, which is used in the present paper as fully synonymous.

7 Arsenijević and Mitić (2016a) provide evidence that the ordering of these constraints depends on other grammatical and semantic properties, such as the agentivity and animacy of the subject.

8 In both investigations, the role of the confound variable is expected to be somewhat reduced for those stimuli in which one of the conjuncts is a NSg noun ending in -*o* (rather than -*e*). In these stimuli, the NSg noun is not syncretic with the FPl noun, which has the ending -*e*. However, since this variable was not controlled, this does not rescue the results of the experiments. It only predicts a somewhat smaller role of the confound variable than if syncretism was full.

9 The fact that verbs with the ending -*o*, the unique NSg ending for the verb, were not produced in Marušič et al. (2015) supports the FPl analysis. Yet, since Arsenijević and Mitić (2016a,b), who conduct an experimental investigation of Sg&Sg conjunctions, do attest the NSg ending -*o* – the fact that it is not attested in Marušič et al. (2015) is probably due to a strong facilitating effect of the Pl conjunct.

10 Participants in both Willer-Gold et al. (2016, 2018) and the present experiment were from the same population: first and second year students (19–23 years old) of non-linguistic majors from the University of Niš who have lived in the area the last 5 years or longer (we only discuss the results from Willer Gold et al. from the University of Niš – which was one among six sites where the experiment was administered).

11 We express our gratitude to the administrators of Ibex Farm, in particular to its author Alex Drummond, for making our work considerably simpler.

12 Frequencies were additionally tested in the SC Word Frequency Corpus (Arsenijević, 2018) with a more contemporary and less formal register-based sample.

13 The five sub-types of fillers were: hybrid agreement nouns with a possessive adjective (*Julijin komšija* “Julija’s neighbor,” where *komšija* can trigger M or F agreement), as in (15b); hybrid agreement nouns with a possessive pronoun (*moje cerekalo* “my laugher,” where *cerekalo* can trigger M or N agreement), as in (15c); NSg animate nouns with an ordinal number [*prvo prase* “(the) first pig,” where *prase* has a hybrid plural form, triggering FSg or NPl agreement], as in (15d); regular agreement nouns with a MSg nominal complement [*uspeh dekana* “(the) success (of the) dean,” where the genitive complement is homonymous with the paucal, and the paucal allows paucal and MPl agreement], as in (15e); regular agreement MSg nouns with a PP complement (*prijatelj iz škole* “the friend from school,” where attraction effects could be expected), as in (15f).

14 The salience of the examples was controlled in the following way. First, one author formulated the examples according to her intuition, and the other evaluated them; where disagreement emerged, examples were replaced and the procedure was repeated for the newly introduced examples.

15 The significance of the difference between agreement patterns within the condition is orthogonal to the hypotheses in the focus of the paper, as they only make predictions about the relative quantities between the conditions, or between the conditions and the base-line ratios. We report these tests in the interest of completeness of the report, as advised by the editors of the volume.

16 We have designed and administered a new experiment along these lines, and we are currently analyzing the data.

## Supplementary Material

TABLE S1 Stimuli and row results of the experiment on the role of plural number and syncretism in conjunct agreement.

## Appendix: on the Choice of the Combination of Gender–Number Combinations

This section clarifies some technical issues about the choices made in the design of our experiment, in light of the special properties of SC morphology. It is aimed primarily for those interested in the theoretical and descriptive linguistic, rather than psycholinguistic aspects of the research.

In our experimental design, we have capitalized on the fact that in SC NSg nouns end either in -*e* or in -*o*, and that their plural ends in -*a*, while at the same time FSg nouns end in -*a*, and their plural forms end in -*e*. This yields a crossed, yet incomplete syncretism.

(19) The crossed incomplete syncretism between F and N nouns in Sg and Pl

**Table:**

	Sg	Pl
N	-e, -o	-a
F	-a	-e

The combination of NSg and FPl allows for the formation of minimal pairs between a syncretic and a non-syncretic pair of nouns, while the combination of NPl and FSg allows for only one possibility, which is syncretic.
(20)

**Table:**

a.	NSg + FPl:	NSg-e & FPl-e vs.	NSg-o & FPl-e
		polje i livade	pismo i olovke
		field.NSg and meadow.FPl	letter.NSg and pen.FPl

b.	NPl + FSg: only	NPl-a & FSg-a
		pisma i olovka
		letter.NPl and pen.FSg

We used minimal pairs as in (20a) in our critical stimuli. The selected option, however, allows for two sub-options, depending on which gender–number combination comes as the first, and which as the last conjunct. This was decided by another similar consideration.

The verb bears the endings: -*o* for NSg, -*a* for NPl and FSg, or -*e* for FPl – i.e., it is possible to distinguish NSg from FPl on the verb. This means that, apart from the unambiguously default masculine ending -*i*, when a verb in -*e* was produced, we were sure that it was FPl, and when a verb in -*o* was produced, we knew that it was NSg. However, when a verb in -*a* was produced – it was uncertain whether it was plural, agreeing in gender with the first conjunct (NPl), or it was singular and agreed in gender with the last conjunct (FSg). The four logically possible combinations and their properties are illustrated in (21).

(21)

**Table:**

NSg	&	FPl	FCA (verb-NPl)	LCA (verb-FPl),	facilitated: LCA in FPl
*-e/-o*		*-e*	*-a*	*-e*	*-e*
FPl	&	NSg	FCA (verb-FPl)	LCA (verb-NPl),	facilitated: FCA in FPl
*-a*		*-e/-o*	*-e*	*-a*	*-a*
NPl	&	FSg	FCA (verb-NPl)	LCA (verb-FPl),	facilitated: FCA in NPl
*-a*		*-a*	*-a*	*-a*	*-a*
FSg	&	NPl	FCA (verb-FPl)	LCA (verb-NPl),	facilitated: LCA in NPl
*-a*		*-a*	*-a*	*-a*	*-e*

It was in the interest of the experiment to minimize the amount of the patterns of agreement realized as the ending *-a*, in order to also minimize the amount of ambiguously interpretable results. Tendencies reported in the literature (one of which is the topic of this paper) – that the verb agrees with the conjunct which bears the plural number and that it rather agrees with the last than with the first conjunct (Marušič et al., 2015; Arsenijević and Mitić, 2016a,b) – imply that if we have a plural conjunct in -*e*, and in particular if it is in the position of the last conjunct, there will be few instances of verbs with the ending -a produced (or even none, as it turned out to be the case).

All combinations other than the selected NSg&FPl would include a considerable participation of the ambiguous ending -*a* on the verb and/or would locate the plural value of number, whose facilitating effects are tested, on the first conjunct – which is a less likely controller of agreement (Willer-Gold et al., 2016, 2018).
